# Gallic acid potentiates the anticancer efficacy of cisplatin in ovarian cancer cells through modulation of the PI3K/AKT/mTOR and CXCL12/CXCR4 signaling pathways

**DOI:** 10.3389/fonc.2025.1653538

**Published:** 2025-09-22

**Authors:** Jinlan Liang, Tingting Lu, Tiyan Shan, HuiTing Liang, Wenjie Wang, Zhijun Song, Yong Tang, Qi Wang

**Affiliations:** 1 Department of Experimental Research, Guangxi Medical University Cancer Hospital, Nanning, China; 2 Key Laboratory of Early Prevention and Treatment for Regional High Frequency Tumor (Guangxi Medical University), Ministry of Education, Nanning, China; 3 University Engineering Research Center of Oncolytic & Nanosystem Development, Guangxi, China; 4 Institute of Life Sciences, Guangxi Medical University, Nanning, Guangxi, China; 5 Guangxi Administration of Traditional Chinese Medicine, Guangxi Botanical Garden of Medicinal Plants, Nanning, Guangxi, China; 6 Department of Urology, Guangxi Medical University Cancer Hospital, Nanning, China

**Keywords:** gallic acid, ovarian cancer, Cisplatin, PI3K/AKT/mTOR signaling pathway, CXCL12/CXCR4 signaling pathways, synergistic effect

## Abstract

**Background:**

This study investigates the antitumor effects of gallic acid (GA) on ovarian cancer cells and its potential synergistic therapeutic effects with cisplatin (DDP) through modulation of the PI3K/AKT/mTOR signaling pathway.

**Methods:**

Systematic evaluations were conducted using both in vitro cell experiments and in vivo animal models to assess the impact of GA alone and in combination with DDP on ovarian cancer cell proliferation, apoptosis, and related signaling pathways.

**Results:**

The results demonstrate that GA significantly inhibits the proliferation of ovarian cancer cells and enhances the anticancer effects of DDP by regulating the PI3K/AKT/mTOR signaling pathway. In in vivo experiments, the combination of GA and DDP significantly inhibits tumor growth and prolongs survival in a mouse model of ovarian cancer without apparent toxicity to vital organs.

**Conclusion:**

This study provides scientific evidence for the potential use of GA as an adjuvant drug in ovarian cancer treatment.

## Introduction

Ovarian cancer is one of the most common malignant tumors in the female reproductive system, ranking tenth in incidence among gynecological malignancies. Due to the lack of effective early diagnostic methods, approximately 70% of patients are diagnosed at an advanced stage. The complex pathogenesis of ovarian cancer and the absence of a cure contribute to its high mortality rate, which is the highest among gynecological malignancies, earning it the moniker “the queen of gynecological cancers” (1–3). Platinum-based chemotherapy drugs are the mainstay treatment for many malignancies, including ovarian cancer, which is typically treated with a combination of surgery and paclitaxel/cisplatin chemotherapy (4, 5). However, studies have shown that while approximately 70% of ovarian cancer patients initially respond well to platinum-based chemotherapy, about 70% develop drug resistance within 2 – 3 years of treatment (6). Once resistance to platinum-based chemotherapy drugs occurs, patient survival rates plummet, with a 5-year survival rate of only around 30% (7). According to data released by the National Cancer Center, the 5-year survival rate for malignant tumors in China has increased from 30.9% a decade ago to 40.5%, while the 5-year survival rate for ovarian cancer has only improved by 0.4%, showing almost no significant progress (8). In current treatment practices, there are virtually no other effective treatment options once patients develop resistance to platinum-based chemotherapy drugs. Therefore, it is imperative to continue searching for new therapies that can significantly improve the survival rate of ovarian cancer patients with minimal side effects.

Although gallic acid (GA) has repeatedly been shown to inhibit ovarian cancer cell proliferation, the reported mechanisms vary and are mostly limited to *in vitro* observations. Borsoi et al (9)demonstrated that GA decreases viability and modulates epigenetic-related genes, yet did not explore any synergy with platinum drugs. He et al. (10)and Sánchez-Carranza et al. (11) focused on anti-angiogenesis or ROS-mediated sensitization to paclitaxel, leaving the interaction with cisplatin unaddressed. Varela-Rodríguez et al. (12) combined GA with myricetin, but lacked mechanistic depth and *in vivo* validation. Collectively, these studies neither clarify whether GA can reverse cisplatin resistance nor elucidate the downstream pathway (CXCL12/CXCR4–PI3K/AKT/mTOR axis) potentially involved. Therefore, the aims of the present study were: 1. To systematically evaluate the synergistic effects of GA in combination with cisplatin in platinum-sensitive and platinum-resistant ovarian cancer models; 2. To identify the key axis (CXCL12/CXCR4-PI3K/AKT/mTOR) driving the synergistic effect; 3. Provide preclinical survival evidence in a syngeneic orthotopic mouse model mimicking human platinum-based relapse.

In recent years, with the deepening research on traditional Chinese medicine, many drugs extracted from natural plants have garnered increasing attention due to their favorable pharmacokinetic properties and mild side effects. Some natural products have been used as alternative cancer therapies or effective adjuvants in chemoradiotherapy (13). For instance, artesunate, a derivative of artemisinin extracted from the plant Artemisia annua, although commonly used for malaria treatment, has been shown to restore the sensitivity of various cancer types to chemotherapy drugs by modulating different signaling pathways (14, 15). Camptothecin, isolated from the tree Camptotheca acuminata, exerts its remarkable anticancer effects by specifically inhibiting the activity of topoisomerase I (16). Curcumin can target the signal transducer and activator of transcription 3 (STAT3) to synergistically enhance the anticancer activity of cisplatin in thyroid cancer cells (17). Ganoderma lucidum polysaccharides inhibit the proliferation of tongue cancer cells by reducing the phosphorylation of EGFR and AKT, thereby inhibiting the EGFR-mediated signaling pathway. This not only enhances cisplatin-induced apoptosis but also ameliorates the cytotoxic effects of cisplatin on normal human oral epithelial cells (18). Natural plant-derived antitumor agents offer unique advantages in reducing adverse reactions, improving quality of life, and decreasing recurrence and metastasis in cancer patients (19).

Gallic acid, also known as pentagalloyl acid, is a natural polyphenolic organic compound widely found in tea, grapes, fruits, and red wine. It has been reported to possess various pharmacological and biological properties, including antibacterial, antiviral, and antitumor activities (20). In recent years, GA has been found to exhibit antitumor effects in different cancer cell lines, including oral, lung, pancreatic, and cervical cancer cells (21). Literature has gradually confirmed that GA has strong proapoptotic activity against various types of cancer (22). GA induces apoptosis and morphological changes in breast cancer cells, suggesting its potential as a natural drug for breast cancer treatment (23). GA can enhance the anticancer effects of cisplatin in non-small cell lung cancer A549 cells via the JAK/STAT3 signaling pathway (24). GA Enhances Cisplatin-induced Death of Human Laryngeal Cancer Cells by Activating the TRPM2 Channel (25).

Zeng et al. (26)found that GA inhibits the proliferation, metastasis, and promotes apoptosis of bladder cancer T24 cells, with its proapoptotic activity closely related to mitochondrial dysfunction and the inhibition of the PI3K/AKT/NF-κB signaling pathway. While GA has been shown to suppress ovarian cancer cells by interfering with PTEN/AKT/HIF-1α (10), ROS/ERK (11) or cell-cycle checkpoints (27), a critical gap remains: its potential to synergize with cisplatin, the first-line drug for ovarian cancer, and the precise signaling axis underlying such synergy remain unexplored. Therefore, this study aims to investigate the antitumor effects of GA on ovarian cancer cells and its potential synergistic therapeutic effects with cisplatin. By integrating transcriptomics, network pharmacology, molecular docking and a clinically relevant orthotopic model, we here define: the optimal concentration window for GA+cisplatin synergy; the CXCL12/CXCR4–PI3K/AKT/mTOR pathway as a mechanistic hub; and prolonged survival without organ toxicity, thereby addressing the therapeutic void left by previous GA-ovarian cancer studies.

## Results and discussion

### Gallic Acid Selectively Inhibits Ovarian Cancer Cell Proliferation and Exhibits a Synergistic Effect with Cisplatin

#### GA inhibits the proliferation of ovarian cancer cells

**Figure 1:**
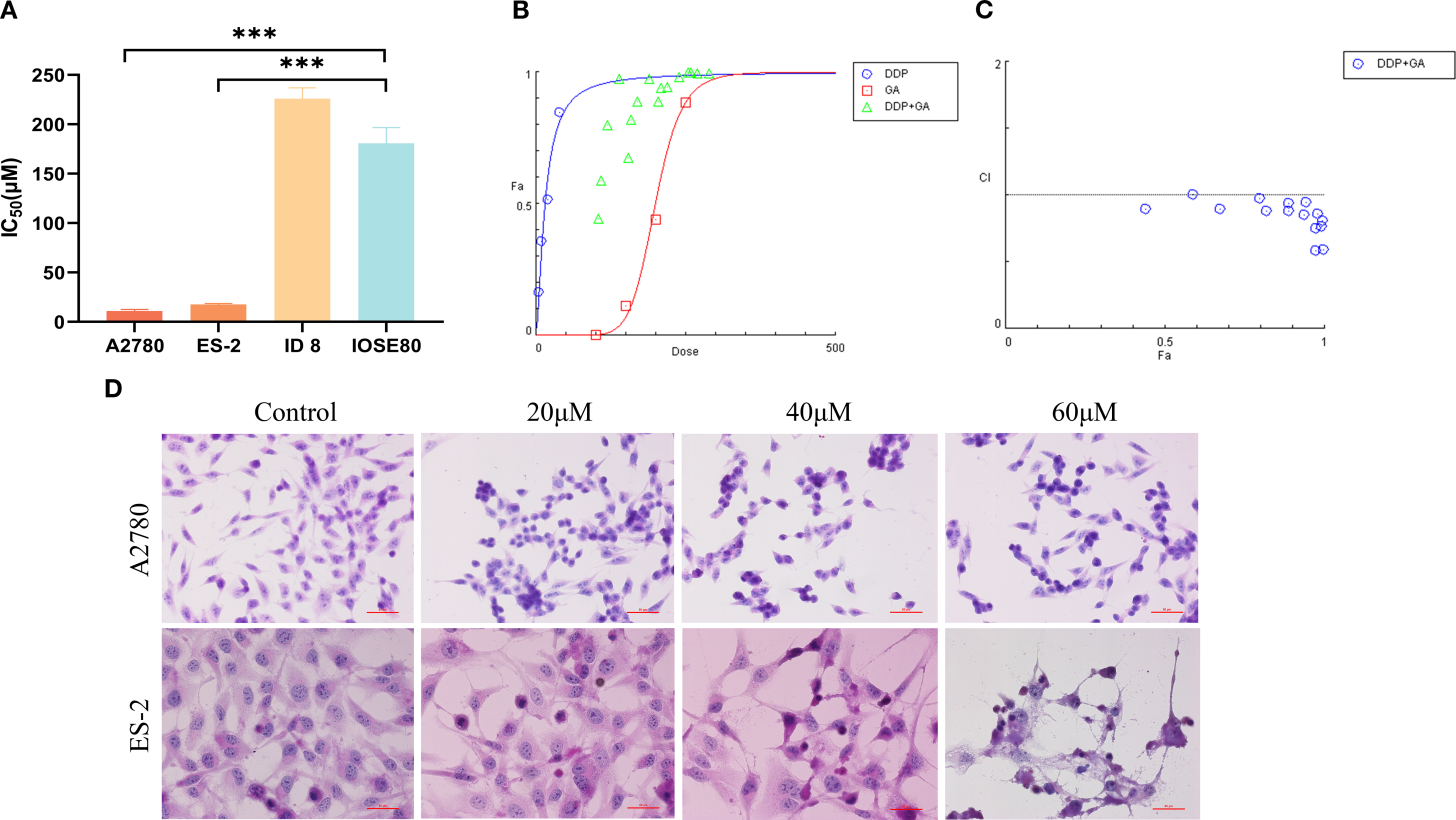
**(A)** IC_50_ of GA on different ovarian cancers at 48h (***P<0.001); **(B)** Dose-response curve of ID8 cells treated with GA combined with DDP; **(C)** CI plot of ID8 cells treated with GA combined with DDP; **(D)** HE staining was used to observe the morphological changes of A2780 and ES - 2 cells treated with GA for 48 hours (scale:50 μm).

CCK-8 assay results (**Table 1**) showed that after 48 hours of GA treatment, the IC_50_ values of A2780 and ES - 2 cells were significantly lower than those of IOSE80 cells (**Figure 1A**), at (10.89 ± 1.77) µM and (17.60 ± 1.12) µM, respectively, with IOSE80 cells having an IC_50_ value of (168.10 ± 12.01) µM (all *P*<0.001). This indicates that GA has cytotoxic effects on A2780 and ES - 2 cells and can significantly inhibit the proliferation of these ovarian cancer cells *in vitro*.

**Table 1:** IC_50_ values of different ovarian cancer cells treated with gallic acid for 48h (μM).

Cell types	IC_50_ value(μM)
A2780	10.89 ± 1.77
ES-2	17.60 ± 1.12
IOSE80	168.10 ± 12.01
ID8	226.00 ± 10.88

#### Synergistic effects of GA and cisplatin

The IC_50_ value of GA for ID8 cells was (226.00 ± 10.88) µM, higher than that of IOSE80 cells. When used in combination with cisplatin, a synergistic effect was demonstrated using CompuSyn software. The fraction affected (Fa) represents the percentage of affected cells (**Figure 1B**), and the combination index (CI) values were all less than 1 (**Figure 1C**), indicating that the combined treatment had a stronger inhibitory effect on cell growth than individual treatments. Therefore, 75 µM and 150 µM of GA in combination with 5 µM of DDP were selected for subsequent experiments. These data indicate that GA significantly reduces proliferation of ovarian cancer cells while sparing non-malignant IOSE80 cells.

#### GA affects the growth of A2780 and ES - 2 ovarian cancer cells

HE staining results (**Figure 1D**) showed that untreated cells had uniform staining and normal morphology. Compared with the untreated group, as the concentration of GA increased, the cell density gradually decreased, cells lost their normal structure, cell volume decreased and became rounder, chromatin became more compact, cytoplasm shrank, nuclei stained darker, cells fragmented, and underwent fragmentation. This indicates that GA can significantly inhibit the growth of A2780 and ES - 2 cells and promote apoptosis.

### Potential targets of GA in ovarian cancer treatment and transcriptome sequencing results

**Figure 2:**
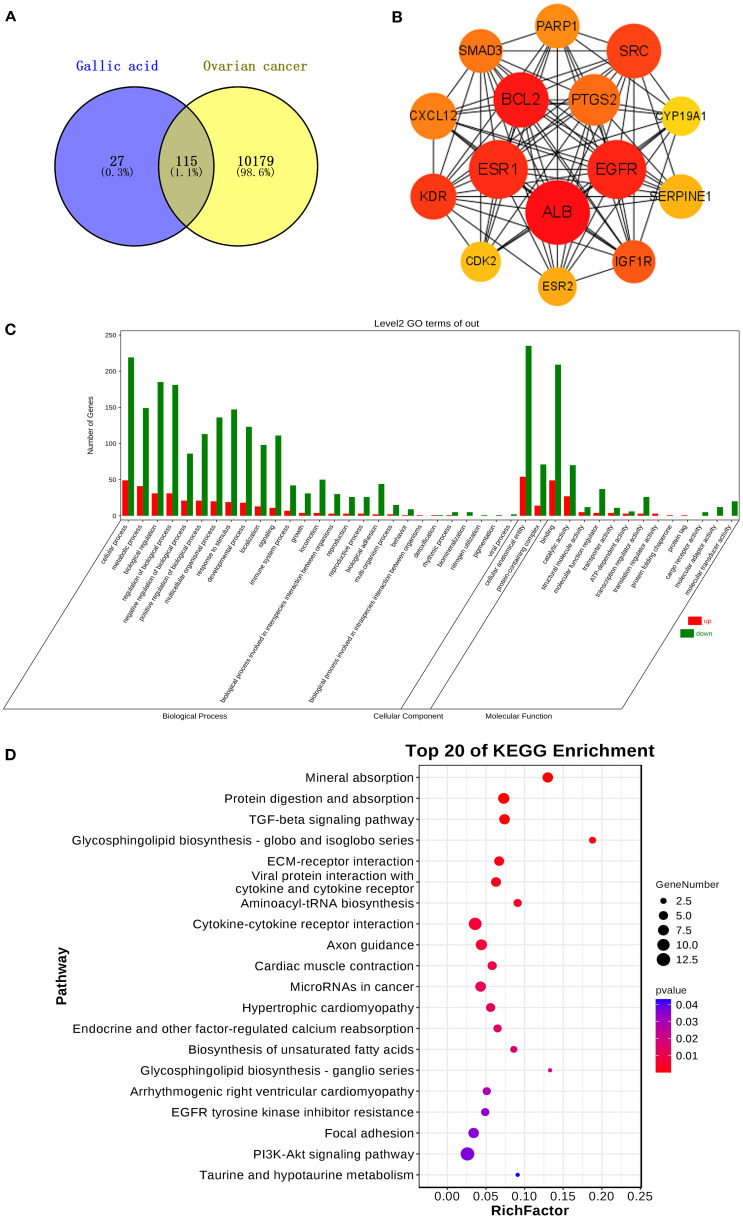
**(A)** Intersection targets of GA and ovarian cancer; **(B)** A core target for ovarian cancer treatment; **(C)** GO enrichment analysis results of ID8 cells after GA treatment; **(D)** Results of KEGG enrichment analysis of ID8 cells after GA treatment.

After intersecting the targets of GA with those of ovarian cancer and removing duplicates, 115 common targets were obtained (**Figure 2A**). A PPI network was constructed, and the top 15 core targets for ovarian cancer treatment were identified as ALB, ESR1, EGFR, SRC, BCL2, SMAD3, PTGS2, PARP1, CDK2, SERPINE1, CYP19A1, CXCL12, KDR, IGF1R, and ESR2 (**Figure 2B**). Molecular docking results showed that GA had strong binding affinity to the core targets of ovarian cancer, with CXCL12 and PARP1 having binding energies less than -6.0 Kcal/mol (28) in three different molecular docking tools, indicating good docking effects (**Table 2**).This suggests that the synergistic mechanism of gallic acid and cisplatin (DDP) may be related to the targeting of key proteins such as CXCL12 and PARP1.

**Table 2:** Molecular docking results for the core targets of GA treatment in ovarian cancer.

Target	PDB protein ID	AutodockVina	CB-dock2	Schrodinger maestro
		Binding energy(Kcal/mol)		
ALB	6YG9	-6.6	-6.3	-5.192
BCL2	8G3S	-6.1	-6.1	-6.328
CDK2	8ERD	-6.2	-5.5	-6.597
CXCL12	4LMQ	-6.3	-6.4	-6.302
CYP19A1	3S79	-6.3	-6.3	-5.998
EGFR	8A2D	-6.1	-5.9	-4.953
ESR1	7QVJ	-6.7	-6	-6.64
ESR2	7XVY	-6.3	-6.3	-4.492
IGF1R	3O23	-5.7	-5.7	-7.278
KDR	3WZE	-6	-6	-6.873
PARP1	5XSR	-6.9	-6.4	-7.573
PTGS2	5IKR	-7	-6.5	-6.59
SERPINE1	7AQF	-6.6	-6.1	-4.709
SMAD3	6ZMN	-6	-6	-4.235
SRC	6TUC	-6.6	-7.2	-6.606

Transcriptome sequencing results were analyzed for trends and differential gene expression using the DESeq2 algorithm, identifying 63 commonly upregulated genes and 308 commonly downregulated genes. GO enrichment analysis revealed that biological processes (BP) in ID8 ovarian cancer cells were mainly enriched in phosphatidylinositol 3-kinase signaling, transforming growth factor-β receptor signaling, apoptosis, and cell proliferation (*P*<0.05); cellular components (CC) were primarily enriched in the extracellular matrix, basement membrane, and plasma membrane regions (*P*<0.05); and molecular functions (MF) were mainly enriched in protein binding, signal receptor binding, and signal receptor activation (*P*<0.05) (**Figure 2C**). KEGG enrichment analysis was primarily focused on the PI3K-Akt signaling pathway, TGF-beta signaling pathway, ECM-receptor interaction, axon guidance, and Rap1 signaling pathway (**Figure 2D**).Transcriptome analysis suggests that GA treatment may inhibit the proliferation and promote apoptosis of ID8 ovarian cancer cells by significantly affecting key signaling pathways such as PI3K-Akt and TGF-β.

### GA enhances the apoptosis of cisplatin on ovarian cancer cells by inhibiting CXCL12/CXCR4 and PI3K/AKT/mTOR signaling pathways

**Figure 3:**
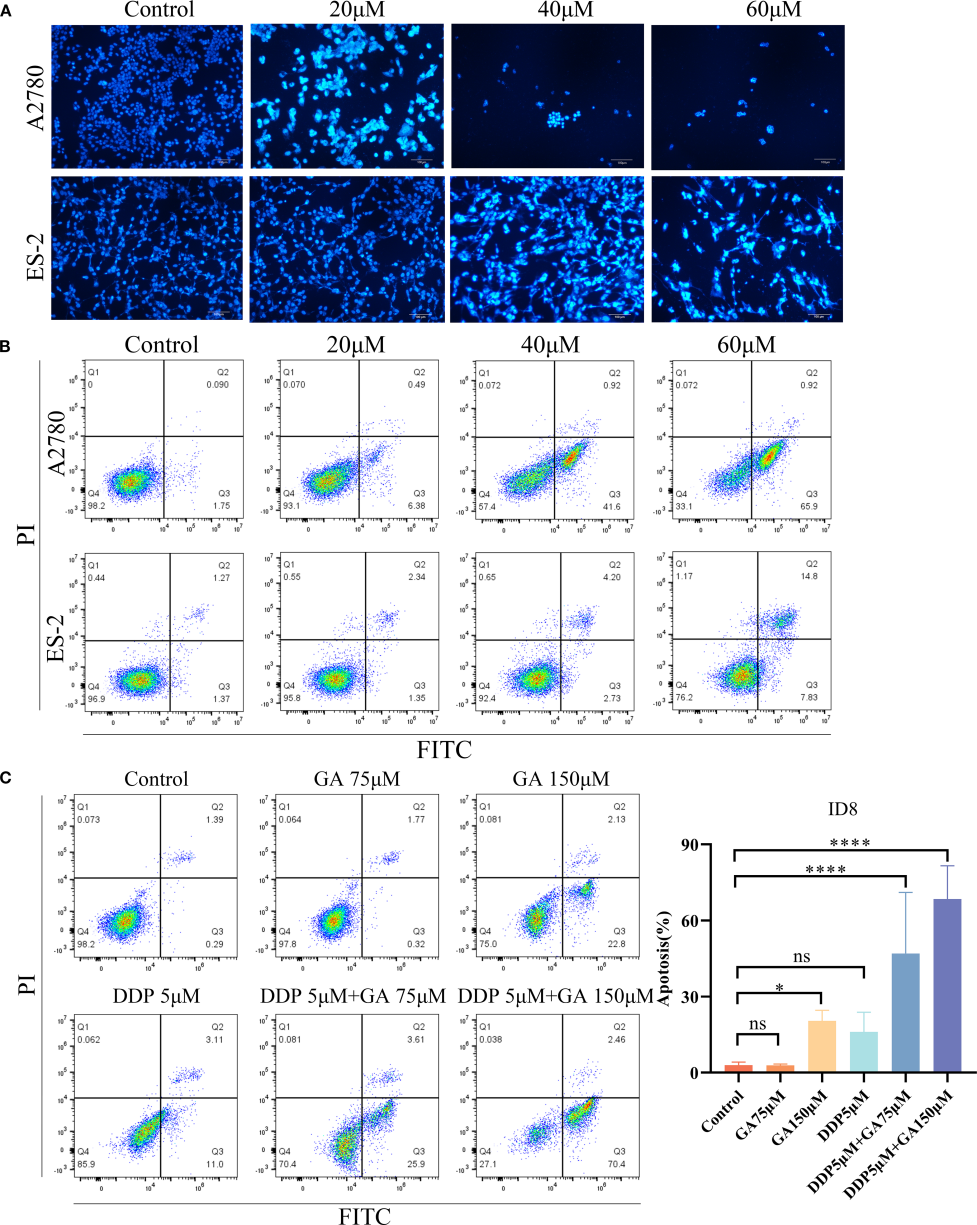
**(A)** Hoechst 33258 staining was used to observe the apoptosis of A2780 and ES - 2 cells treated with GA for 48h (scale:100 μm); **(B)** Apoptosis of ovarian cancer A2780 and ES - 2 cells treated with different concentrations of GA for 48h; **(C)** Apoptosis of ID8 cells treated with GA alone, DDP alone or in combination for 48h.

Hoechst 33258 staining results (**Figure 3A**) showed that compared with the control group, the blue fluorescence intensity of A2780 and ES - 2 cells increased with the concentration of GA, indicating an increase in apoptotic cells and a decrease in cell numbers. This demonstrates that GA can effectively induce apoptosis in ovarian cancer A2780 and ES - 2 cells.

Flow cytometry results (**Figures 3B, C**) showed that compared with the untreated group, the apoptosis rates of A2780 and ES - 2 cells treated with GA (20, 40, 60 µM) for 48 hours significantly increased (all *P*<0.01) and rose with increasing drug concentration. For ID8 cells, the apoptosis rate was low when treated with GA or cisplatin alone, but when treated with the same concentration in combination, the apoptosis rate significantly increased and rose with increasing drug concentration, with rates of 47.02% and 67.42%, respectively. This indicates that the combination of GA and cisplatin can produce a significant synergistic effect in inducing apoptosis in ovarian cancer cells.

**Figure 4:**
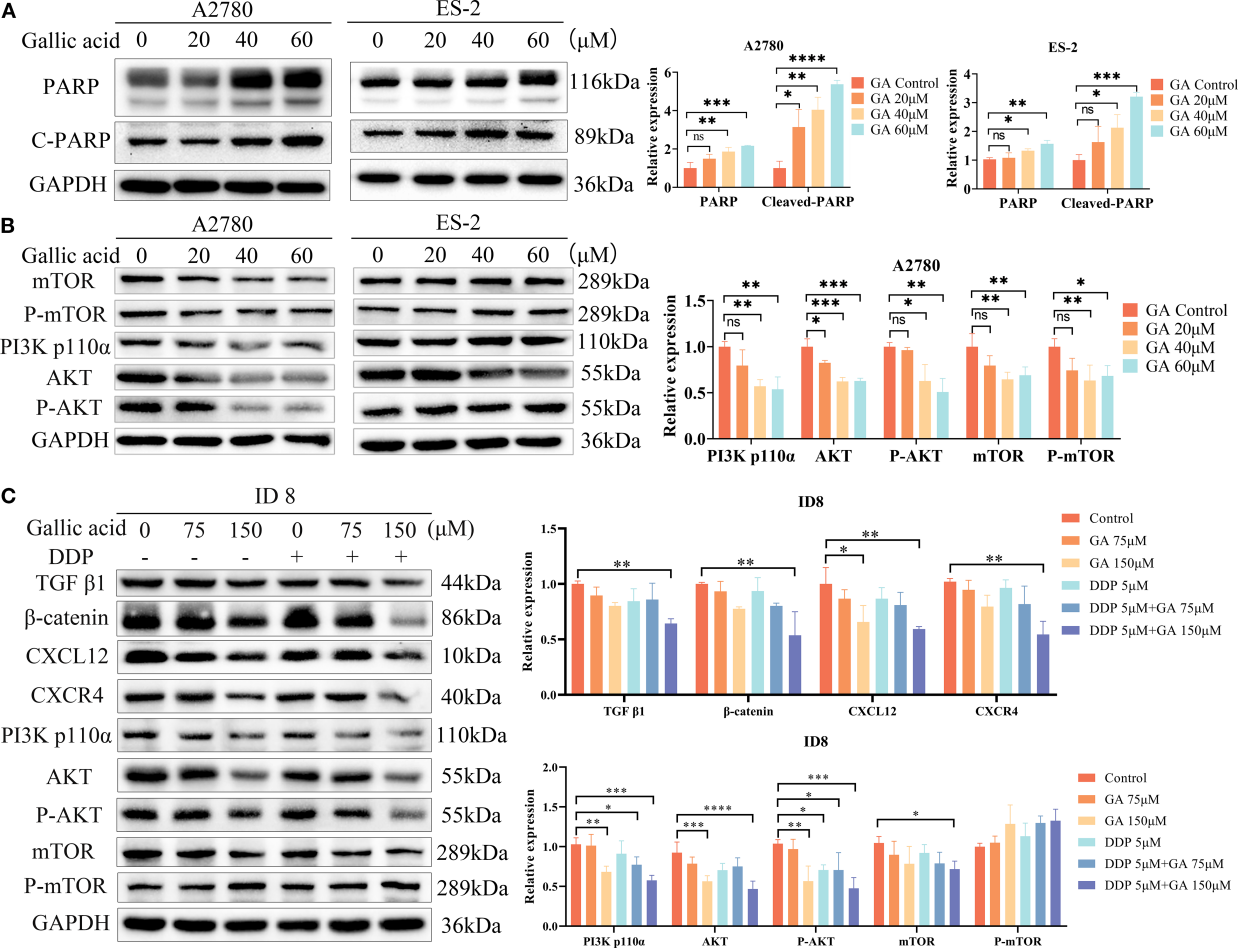
**(A)** Expression levels of apoptosis-related proteins in ovarian cancer A2780 and ES - 2 cells treated with GA for 48h; **(B)** Effect of GA on the expression levels of PI3K/Akt/mTOR signaling pathway related proteins in ovarian cancer A2780 and ES - 2 cells after 48h treatment; **(C)** Expression levels of CXCL12/CXCR4 and PI3K/Akt/mTOR signaling pathway related proteins in ovarian cancer ID8 cells treated with GA and DDP for 48 hours (****P< 0.0001, ***P< 0.001, **P<0.01, *P<0.05, ns indicated no statistically significant difference).

Further Western blot analysis of apoptosis-related proteins revealed that compared with the untreated group, after 48 hours of GA treatment, the expression levels of PARP and Cleaved PARP proteins increased in A2780 and ES - 2 cells (*P*<0.05) (**Figures 4A, B**). In A2780 cells, the expression of PI3K p110α, AKT, P-AKT, mTOR, and P-mTOR was inhibited, and this trend became more pronounced with increasing GA concentration. In ES - 2 cells, the expression of PI3K/AKT/mTOR pathway-related proteins was not inhibited. In ovarian cancer ID8 cells, compared with the control, GA-only, and DDP-only groups, the expression of CXCL12 and CXCR4 was significantly inhibited in the combination treatment group, and the expression of downstream PI3K, AKT, P-AKT, and mTOR was also inhibited, with this trend becoming more pronounced with increasing GA concentration. The expression of phosphorylated mTOR did not change significantly. Additionally, the expression of TGF-β1 and β-catenin proteins was also significantly reduced in the combined GA and DDP treatment (**Figure 4C**). In summary, this study confirmed that gallic acid can effectively induce apoptosis of ovarian cancer cells and synergize with cisplatin. Its molecular mechanism is cell-type-specific and is achieved in A2780 and ID8 cells by inhibiting CXCL12/CXCR4 and PI3K/AKT/mTOR pathway signaling, while in ES - 2 cells it functions in other ways that do not rely on this pathway.

### C57BL/6J mouse xenograft model results

#### Monotherapy with GA and combination therapy with cisplatin inhibit tumor growth in mice

**Figure 5:**
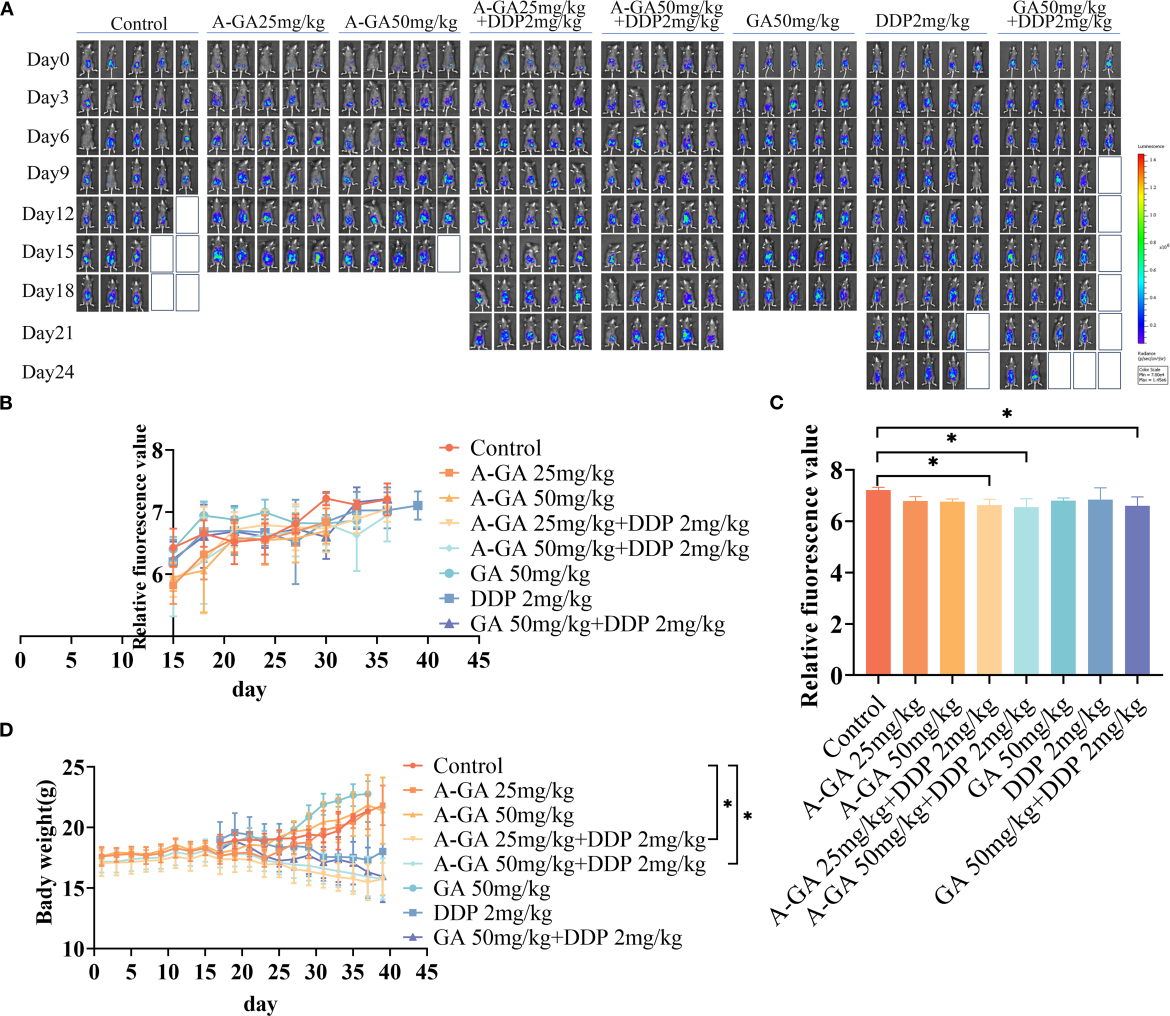
**(A)** Fluorescence expression in the intraperitoneal model of mouse ovarian cancer xenografts after GA monotherapy, DDP monotherapy, and combination therapy; **(B)** The changes of fluorescence value in the intraperitoneal model of mouse ovarian cancer xenograft after GA monotherapy, DDP monotherapy and combination therapy; **(C)** Comparison of fluorescence values in the intraperitoneal model of mouse ovarian cancer xenografts on the 30th day after modeling; **(D)** Changes in body weight of mouse ovarian cancer xenograft model after GA monotherapy, DDP monotherapy and combination therapy. *P< 0.05.

From the time of tumor modeling, the body weight of mice was measured every two days, the survival status of mice was monitored, and *in vivo* imaging was performed every three days to observe changes in abdominal tumors (**Figures 5A-C**). The results showed that regardless of whether GA pretreatment was applied after modeling, tumors appeared 15 days after modeling. The fluorescence values of the control group mice continued to increase after tumor formation, reaching a maximum on day 15, accompanied by ascites and significant weight gain. The fluorescence values of the treatment groups were delayed in reaching their maximum values, and the DDP and combination therapy groups showed delayed ascites symptoms in the mice after treatment (**Figure 5D**).

#### GA combined with cisplatin prolongs survival in mice

**Figure 6:**
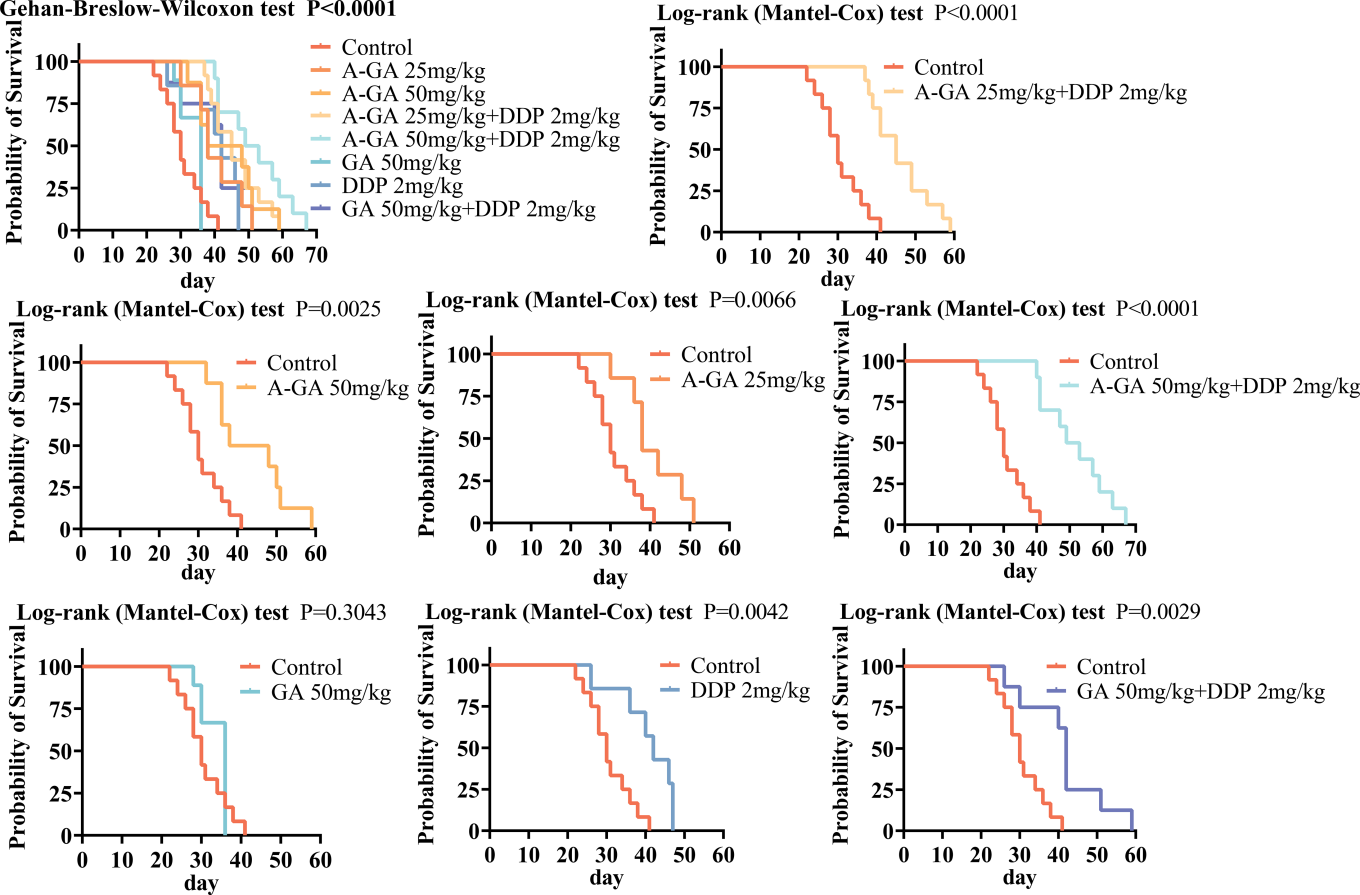
Survival comparison of GA monotherapy, DDP monotherapy, and combined therapy in a syngeneic xenograft model of ovarian cancer in mice (n=6).

Log-rank tests were used to analyze the survival of mice in the Control, A-GA, A-GA+DDP, GA, DDP, and GA+DDP groups. The results (**Table 3**) showed that the median survival times were 30 days for the Control group, 38 days for the A-GA 25 mg/kg group, 43 days for the A-GA 50 mg/kg group, 45 days for the A-GA 25 mg/kg + DDP 2 mg/kg group, 51 days for the A-GA 50 mg/kg + DDP 2 mg/kg group, 36 days for the GA 50 mg/kg group, 42 days for the DDP 2 mg/kg group, and 42 days for the GA 50 mg/kg + DDP 2 mg/kg group. Although the median survival time of the GA group was not statistically different from that of the control group, the survival time was still significantly prolonged. Except for the GA group, the survival times of the other groups were significantly different from that of the control group (*P*<0.05) (**Figure 6**). Mice treated with GA immediately after modeling had longer survival times than those treated with GA after tumor formation. Under the premise of ensuring a safe dosage, the higher the drug dosage, the longer the survival time. Mice treated with 50 mg/kg of GA after modeling did not have an extended tumor formation time, but mice treated with 2 mg/kg of DDP in combination after tumor formation had longer tumor-bearing survival times than those treated with the same dosage of DDP alone or the same dosage of GA and DDP in combination. This indicates that GA can prolong the survival of mice (**Figure 6**). It can be inferred that GA is a mild antitumor active component, whose onset and effect are slower than those of DDP. It plays an auxiliary therapeutic role in the body and effectively enhances the action of DDP.

**Table 3:** Median survival time of homologous subcutaneous tumor model of mouse ovarian cancer.

Groups	Mean	Median survival(day)
Control	30.67 ± 5.694	30
A-GA 25mg/kg	40.43 ± 7.208	38**
A-GA 50mg/kg	43.75 ± 9.513	43**
A-GA 25mg/kg+ DDP 2mg/kg	46.08 ± 7.403	45***
A-GA 50mg/kg+ DDP 2mg/kg	51.70 ± 9.661	51***
GA 50mg/kg	33.78 ± 3.383	36
DDP 2mg/kg	40.57 ± 7.613	42**
GA 50mg/kg+ DDP 2mg/kg	41.50 ± 10.50	42**

Compared with the control group, **:*P*<0.01; * * *: *P* < 0.001.

#### HE staining results show the safety of GA on vital organs *in vivo*

**Figure 7:**
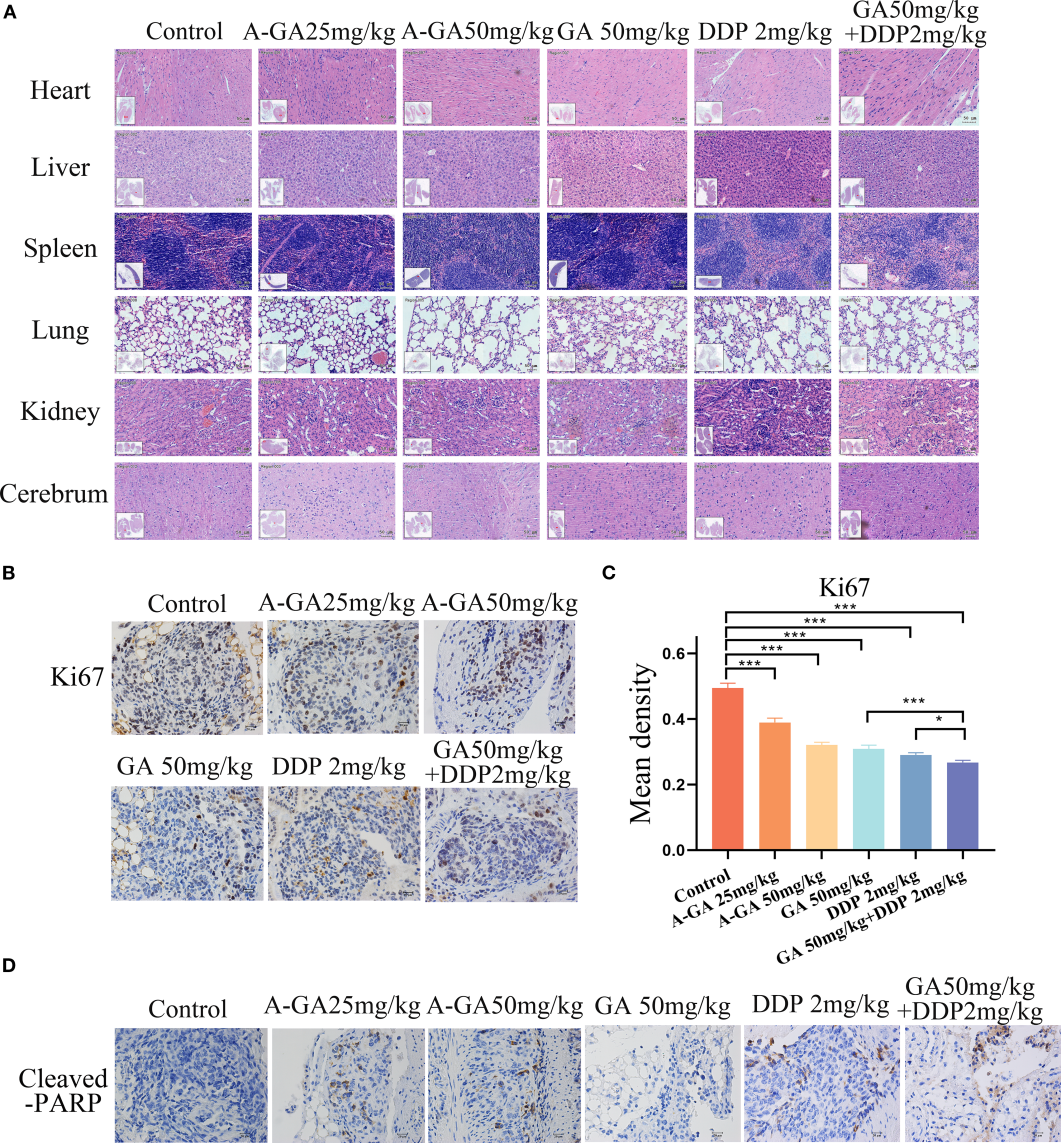
**(A)** HE staining was used to observe the important organs and histomorphological morphology of the transplanted tumor in the intraperitoneal model of mice with ovarian cancer after treatment with GA alone, DDP alone or in combination (scale:50 μm); **(B)** Immunohistochemistry was used to detect the expression of Ki67 in mouse ovarian cancer xenografts (scale:20 μm); **(C)** Quantitative analysis of Ki67-positive cells in xenograft tissues. Data are presented as mean ± SD; ***P < 0.001, *P < 0.05. **(D)** Immunohistochemical detection of Cleaved-PARP expression in mouse ovarian cancer xenograft tissues (scale:20 μm).

The vital organs of mice (heart, liver, spleen, lungs, kidneys, and brain) were fixed, embedded, and sectioned. HE staining was performed and the histopathological changes of the transplanted tumor were observed under an optical microscope. The results (**Figure 7A**) showed that compared with the control group, no obvious abnormalities were observed in the vital organs of mice in the treatment groups. Previous studies have found that the LD50 of intraperitoneal injection of GA in mice is 4.3 g/kg, and that of cisplatin is 27 mg/kg. This proves that the dose of GA used in this experiment has minimal toxicological effects on vital organs *in vivo*.

### IHC validation of the inhibition of Ki67 expression and promotion of cleaved-PARP expression by GA combined with cisplatin *in vivo*

Immunohistochemical staining was used to detect the expression of Ki67 and Cleaved-PARP in the xenograft tumor tissues of C57BL/6J mice with ovarian cancer. Positive staining for Ki67 and Cleaved-PARP was located in the nuclei. The staining results (**Figures 7B, C**) showed that after GA treatment, the expression of Ki67 in the xenograft tumor tissues of ID8 cells decreased, and the expression of Ki67 in the combination treatment group of GA and DDP was significantly lower than that in the single GA and single DDP groups, with statistically significant differences (P<0.001). Compared with the control group, the expression of Cleaved-PARP in the xenograft tumor tissues of ID8 cells increased in the treated group (**Figure 7D**), indicating that GA can effectively inhibit ovarian cancer proliferation *in vivo*, and its combination with DDP can produce a synergistic effect.

Ovarian cancer remains the most lethal gynecological malignancy largely because of rapid acquisition of platinum resistance. Active monomers and secondary metabolites extracted from natural plants are important sources for the development of new drugs and have unique advantages in the treatment of malignant tumors (29). While gallic acid (GA) has repeatedly been reported to inhibit ovarian cancer cell proliferation (9–11), the majority of these studies were confined to *in vitro* cytotoxicity or single-pathway descriptions. The present work provides the first integrated demonstration that GA markedly sensitizes platinum-resistant ovarian cancer to cisplatin by rewiring the CXCL12/CXCR4-PI3K/AKT/mTOR axis and translates this mechanism into prolonged survival in an immune-competent orthotopic model.

The PI3K/AKT/mTOR signaling pathway is a key intracellular signaling pathway closely related to tumor cell proliferation, apoptosis, angiogenesis, metastasis, and resistance to radiotherapy and chemotherapy. Dong et al. (30) found that upregulation of the PI3K/AKT pathway is a potential indicator of lack of response to neoadjuvant chemotherapy in stage II/III breast cancer patients. Jin et al. (31) identified through whole-exome sequencing and mass spectrometry-based proteomics that PI3K/AKT signaling pathway-related genes are upregulated in small cell lung cancer tissues, suggesting that PI3K/AKT pathway activation may be a potential mechanism of chemoradiotherapy resistance in small cell lung cancer.

Active monomers from medicinal plants can significantly enhance the anticancer effects of chemotherapeutic drugs by inhibiting the PI3K/AKT/mTOR pathway. Di et al. (32) demonstrated that Ailanthone increases cisplatin-induced apoptosis and autophagy in non-small cell lung cancer cells via PI3K/AKT/mTOR. Curcumin can inhibit the PI3K/AKT and JAK/STAT3 pathways in ovarian cancer cells to enhance the anticancer effects of cisplatin (33). Jiang et al. (34) found that Fisetin enhanced cisplatin sensitivity in renal cell carcinoma through the CDK6/PI3K/Akt/mTOR signaling pathway. Fucoidan Enhances Cisplatin-induced Effects on SCC - 25 Human Oral Cancer Cells by Inhibiting the PI3K/AKT Pathway (35). GSK3 (glycogen synthase kinase 3) is a highly conserved serine/threonine kinase that plays an important role in the degradation of cytoskeletal protein β-catenin and apoptosis (36). After AKT phosphorylates GSK3, GSK3 is inactivated, leading to the accumulation of β-catenin in the cytoplasm and its translocation into the nucleus, activating genes related to cell division and growth regulation. Concurrently, AKT activation accelerates glycolysis, increases ATP production, and inhibits apoptosis (37). Therefore, GA inhibits AKT, thereby reversing the inactivation of GSK3 and promoting β-catenin degradation, thereby enhancing the apoptotic effects of cisplatin on ovarian cancer cells.

CXCL12 (CXC chemokine ligand 12) belongs to the CXC chemokine family and plays multiple roles in cell homing and tumor metastasis (38). CXCR4 (CXC chemokine receptor 4) is the specific receptor for CXCL12. The binding of CXCL12 and CXCR4 is specific and one-to-one, hence the term CXCL12/CXCR4 axis. This axis is involved in various pathological and physiological processes, including intercellular communication, immune response mediation, angiogenesis, and tumor invasion and metastasis (39, 40). Yin et al. (41) found that CXCL12 can activate the serine/threonine kinase AKT through autocrine or paracrine signaling, leading to the degradation of collagen fibers, disruption of the basement membrane, and induction of tumor cell proliferation, migration, and invasion. Clearly, the PI3K/AKT/mTOR signaling pathway is one of the downstream pathways of the CXCL12/CXCR4 axis and plays an important role in tumor metastasis and angiogenesis. Activated PI3K generates PIP3, which promotes AKT phosphorylation and translocation to the nucleus, releases the inhibitory effect of mTOR on the inhibitory protein, activates TORC1, promotes mTOR phosphorylation, and thereby mediates cell proliferation and exerts oncogenic functions (42).

AKT hyper-phosphorylation is now recognized as a core driver of platinum efflux, enhanced DNA-damage repair and anti-apoptotic signaling in ovarian cancer (43). Yet direct PI3K or AKT inhibitors have been limited by on-target metabolic toxicities. We discovered that GA does not directly bind PI3K catalytic subunits (**Table 1**); instead, it down-regulates CXCL12 transcription, thereby disrupting the CXCL12/CXCR4 autocrine loop that normally maintains PI3K membrane recruitment and AKT Ser473 phosphorylation. This mode of action is consistent with findings in these breast cancers where CXCR4 blockade restored sensitivity to PI3K inhibitors (44, 45), but has never been studied in ovarian cancer and has never been associated with GA.

Earlier reports showed GA could inhibit proliferation via ROS/ERK (11) or PTEN/AKT/HIF-1α (10); however, none examined cisplatin synergy or survival extension. Here, CompuSyn analysis revealed strong synergy (CI < 0.7) at clinically achievable GA concentrations (75 – 150 μM), and—critically—median survival increased from 30 to 51 days in the ID8 peritoneal model (**Figure 6**). This is the first *in vivo* evidence that GA converts a platinum-refractory phenotype into a platinum-responsive state.

Multi-omics integration highlights GA as an epigenetic-immune-metabolic modulator. Through network pharmacology, molecular docking and transcriptome sequencing, transcriptome analysis identified 308 down-regulated genes enriched in PI3K-Akt and TGF-β pathways. Pathway interaction analysis (Cytoscape 3.9.1) identified CXCL12 as a major regulator and its inhibition inhibited both MTOR-driven glycolysis (HK2, LDHA downregulation) and TGF-β-mediated EMT (SNAI1, VIM inhibition). This pleiotropy is consistent with the 2024 single-cell Atlas of high-grade serous ovarian Cancer, which identified CXCL12 high tumor cells as a platinum-resistant glycolytic subset (46). The key pathways of GA in the treatment of ovarian cancer may be CXCL12/CXCR4 axis and PI3K/AKT/mTOR signaling pathway. Based on the *in vitro* experiments, the anti-ovarian cancer cell mechanism of GA was explored, and it was confirmed that GA may inhibit cell proliferation and induce cell apoptosis by regulating the PI3K/AKT/mTOR related pathway. The combination of GA and DDP also achieved good combined anti-tumor effect. The combination of GA and DDP promoted the apoptosis of ovarian cancer cells induced by DDP, and also jointly regulated the CXCL12/CXCR4 axis and PI3K/AKT/mTOR related pathways. In addition, it may regulate TGF-β1 and β-catenin related proteins to exert anti-ovarian cancer activity. It is worth noting that GA did not effectively reduce the expression level of p-mTOR in this study, suggesting that there is a compensatory mechanism within the PI3K/Akt/mTOR pathway to maintain its phosphorylation state (47). When Akt is strongly inhibited, negative feedback inhibition of upstream signals (such as receptor tyrosine kinase RTKs) or parallel pathways (such as MAPK pathways) may be undone, resulting in compensatory signaling activation to maintain mTOR phosphorylation. This means that although the total mTOR pool is reduced, the proportion of remaining mTOR molecules “overactivated” by this compensatory signal may have increased, maintaining the level of p-mTOR. The most critical conclusion should be based on the phosphorylation state of the downstream effector molecule (S6K, 4E-BP1).

.*In vivo* experiments, it was found that GA had a slow onset of action, and the combined use of GA with DDP took a longer time to show a combined effect. A longer period of use of GA could promote the apoptotic effect of DDP and significantly prolong the survival time of mice. GA is widely found in plants such as Cornus officinalis, Toxicodendron vernicifluum, Quercus cortex, and tea, with mature extraction processes and low production costs. Moreover, it exhibits extremely low toxicity *in vivo* (48, 49). Therefore, GA can be used clinically as an adjuvant treatment after surgery or as an adjuvant dietary therapy. Long-term postoperative use of GA may help extend the recurrence-free and survival times of patients.

In summary, the active monomer from medicinal plants, gallic acid, exhibits significant anti-ovarian cancer activity with multi-target and multi-pathway characteristics. It can enhance the efficacy of platinum-based drugs, and the combination with platinum-based drugs is a promising anticancer regimen. GA is abundant in dietary sources and has an established safety profile (mouse LD_50_ 4.3 g/kg). At 50 mg/kg—only 1/86 of the LD_50_—GA achieved robust efficacy without weight loss or organ toxicity. In patients, equivalent plasma levels (~5 µM free GA) are attainable with 1 – 1.5 g oral supplementation, suggesting feasibility for adjuvant maintenance therapy after cytoreductive surgery. Although the specific mechanisms of action require further research, gallic acid, as a natural plant active monomer, holds promise as a new adjuvant drug for ovarian cancer treatment. However, there are still many shortcomings in this study, such as the ID8 allograft tumor model can better simulate the process of platinum-resistant recurrence, there is still a lack of validation of human xenograft tumors (PDX) and organoid models. Although intraperitoneal administration of mice mimics intraperitoneal chemotherapy, the oral bioavailability of GA, the maximum blood concentration in humans, and the potential drug-drug interaction (DDI) with cisplatin still need to be confirmed by clinical trials. The upstream regulatory mechanism of GA on the CXCL12/CXCR4 axis and the downstream effects of the PI3K/AKT/mTOR pathway have not yet.

This study systematically evaluated the antitumor effects of gallic acid (GA) on ovarian cancer cells and its potential synergistic therapeutic effects with cisplatin (DDP) through *in vitro* and *in vivo* experiments. The results demonstrated that GA significantly inhibits the proliferation of ovarian cancer cells and enhances the anticancer effects of DDP by modulating the PI3K/AKT/mTOR signaling pathway. In *in vivo* experiments, the combination of GA and DDP significantly inhibited tumor growth and prolonged survival in a mouse model of ovarian cancer without apparent toxicity to vital organs. This study provides scientific evidence for the potential use of GA as an adjuvant drug in ovarian cancer treatment, warranting further exploration of its clinical application potential. Although previous studies have reported that GA alone can inhibit the proliferation of ovarian cancer cells, the mechanism of CXCL12/CXCR4-PI3K/AKT/mTOR axis mediated cisplatin sensitivity and survival benefit have not been reported in public. This study reveals the potential of GA in reversing cisplatin resistance for the first time. CXCL12/CXCR4-PI3K/AKT/mTOR signaling pathway significantly enhanced the efficacy of platinum-based chemotherapy. Through the systematic integration of transcriptome, molecular docking and mouse survival model, this study significantly expands the theoretical basis and translational prospects of GA in the treatment of ovarian cancer, provides a promising clinical supplement for patients who fail to receive platinum therapy, and provides a reasonable basis for biomarks-driven clinical trials of GA combined with standard platinum chemotherapy.

## Experimental section

### General experimental procedures

Ovarian cancer A2780 and ES - 2 cells were purchased from the National Biomedical Experimental Cell Resource Bank. ID8 cells were donated by the Department of Obstetrics and Gynecology, Peking University People’s Hospital. Normal ovarian IOSE80 cells provided by Medical Experimental Center, Institute of Life Science, Guangxi Medical University. RPMI1640 medium, McCoy ‘5A medium, DMEM high glucose medium, fetal bovine serum, and 0.25% trypsin containing EDTA were purchased from Corning, USA. Other reagents and consumables related to this experiment were mainly purchased from Thermo Fisher Scientific (USA), Beyotime Institute of Biotechnology (China), and Solarbio Science & Technology Co., Ltd. (China). Gallic acid was synthesized and purified and generously provided by Professor Zhijun Song. The instruments used were provided by the Laboratory of the Affiliated Tumor Hospital of Guangxi Medical University.

### Cell culture

A2780 cells were cultured in RPMI1640 medium (10 - 040-CVRC, Corning, USA), ES - 2 cells in McCoy’s 5A medium (12330031, Corning, USA), and ID8 and IOSE80 cells in high-glucose DMEM medium (10 - 013-CVRC, Corning, USA). Complete medium was prepared by adding 10% fetal bovine serum (35 - 081-CV, Corning, USA), 1% penicillin-streptomycin solution (C0224, Beyotime Institute of Biotechnology, China), and 1% L-glutamine (ST025, Beyotime Institute of Biotechnology, China). Cells were maintained in a humidified incubator at 37°C with 5% CO2, with medium changes every 2 – 3 days. When the cell density reached approximately 90%, cells were digested with 0.25% trypsin containing EDTA (25 - 053-CI, Corning, USA) and passaged at a 1:3 ratio. Exponentially growing cells in good condition were used for experiments.

### Gallic acid purity determination

Accurately weigh 4.63 mg of gallic acid standard (batch number: 11831 - 201906, China Institute of Food and Drug Control, the content is calculated as 91.5%), put it in a 10 ml volumetric flask, add chromatography methanol to dissolve, dilute to the scale, shake well, and obtain a control solution with a concentration of 0.426 mg/ml. Accurately measure 0.2, 0.6, 1.0, 1.8 and 2.2 ml of the above control solutions in a 10ml volumeic flask, add chromatographic methanol to dilute to the scale, and shake well, that is, the gallic acid series control solution with concentrations of 8.48, 25.44, 42.40, 76.72 and 93.28 μg/ml respectively is obtained. 1 μl of each standard solution was precisely measured, injected into a liquid chromatograph (Shimadzu LD - 40D ultra-high performance liquid chromatograph, Shim-pack GIST HSS-C18 (2.1×100 mm, 2μm) column), and the chromatographic conditions were 0.05% phosphoric acid solution-methanol (93:7) as the mobile phase, and elution at the same degree for 12 minutes; Injection volume: 1μl; Column temperature: 35 °C; Wavelength: 271 nm, record chromatogram. The standard curve of gallic acid was calculated by peak area according to the external standard method. Take gallic acid for the test (provided by Guangxi Medicinal Botanical Garden) 0.26 mg, weigh it precisely, put it in a 10 ml measuring flask, add chromatographic methanol to dissolve and dilute to the scale, shake well, filter with a 0.22μm needle filter, and take the filtrate as the test solution. 1 μl of each of the test solution was precisely measured, injected into a liquid chromatograph, and performed three times in parallel, recording the chromatographic area, substituting the regression curve equation, and calculating the purity of the sample to be 98.34%.

### CCK-8 assay for assessing the cytotoxic effects of GA and synergistic effects with cisplatin

Logarithmically growing A2780, ES - 2, and IOSE80 cells were adjusted to a density of 5×10^4 cells/mL and seeded at 100 µL per well in 96-well plates. After 24 hours, the medium was removed, and GA powder (provided by Guangxi Botanical Garden, purity: HPLC>98.34%) was dissolved in DMSO (D8371, Beijing Solarbio Science & Technology Co., Ltd., China) to prepare a 250 mM GA stock solution. This solution was then diluted with the corresponding complete medium to different concentrations. The 96-well plate was filled with the diluted solutions, and the control group received 100 µL of complete medium per well, with five replicates per group. After 48 hours of incubation, the medium was removed, and 100 µL of complete medium containing 10% CCK - 8 solution (CK04, DOJNDO, Japan) was added to each well. The plate was incubated in the incubator for another 2 hours, and the absorbance at 450 nm was measured using a microplate reader (Synergy H1, BioTek, USA). The cell inhibition rate was calculated as follows: (*ODcontrol*​−*ODblank*​)(*ODcontrol*​−*ODtreated*​)​×100%. The IC_50_ values were determined, and the experiment was repeated three times.

For ID8 cells, the density was adjusted to 5×10^4 cells/mL, and 100 µL was seeded per well in 96-well plates. After 24 hours, the medium was discarded and replaced with 100 µL of complete medium containing different concentrations of GA and cisplatin. The experimental groups were set with four concentrations of GA (100 µM, 150 µM, 200 µM, and 250 µM) and four concentrations of cisplatin (5 µM, 10 µM, 20 µM, and 40 µM) for combination. Negative control and blank groups (both containing 1‰ volume fraction of DMSO) were also established, with three replicates per group. After 48 hours of incubation, the medium was discarded, and 100 µL of complete medium containing 10% CCK - 8 reagent was added to each well. The plate was incubated in the incubator for 2 hours, and the OD values at 450 nm were measured. The cell inhibition rates were calculated, and the combination index (CI) (50)was analyzed using CompuSyn software.

### HE staining to observe the morphology of A2780 and ES - 2 cells

Logarithmically growing A2780 and ES - 2 cells were digested and counted, and the density was adjusted to 1×10^5 cells per well and seeded onto cover slips (placed in 6-well plates). After 24 hours of incubation, the medium was replaced with complete medium containing different concentrations (0, 20, 40, and 60 µM) of GA and continued to be cultured for 48 hours. The cover slips were then removed, fixed in 95% ethanol for 20 minutes, stained with hematoxylin (C005S, Beyotime Institute of Biotechnology, China) for 8 minutes, followed by eosin staining for 1 minute, and mounted with glycerin gelatin mounting medium (C0187, Beyotime Institute of Biotechnology, China). The morphological changes of the cells were observed under a microscope (Nikon, Japan) and photographed. This was similar to the IC50 of Bo et al. (51) in lung cancer Calu-cell GA in the range of 10 to 50 μM, and consistent with the significant morphological changes produced by He et al. (10) in ovarian cancer at 40 μM.

### Network pharmacology and molecular docking to predict targets of GA in ovarian cancer treatment

The targets of GA were predicted using PubChem, Swiss Target Prediction, and SEA databases, and ovarian cancer-related targets were retrieved from the GeneCards database using “ovarian cancer” as the keyword (all searches were conducted on January 29, 2024). The intersection of GA targets and ovarian cancer-related targets was obtained and imported into the STRING database to construct a protein-protein interaction (PPI) network. The PPI network was then imported into Cytoscape 3.9.1 software, and core targets were identified using the MCC algorithm (52) of the Cyto Hubba plugin. The 3D structure of GA was downloaded from the PubChem database as the ligand, and the corresponding target protein structures were downloaded from the PDB protein database as the receptors. Molecular docking was performed using AutoDockVina 1.1.2 software, Schrodinger Maestro 13.5 software, and the CB-dock2 online molecular docking tool (https://cadd.labshare.cn/cb-dock2/index.php). The ligands and receptors were processed for dehydration and hydrogen addition before docking. The results were visualized using PyMOL software.

### Transcriptome sequencing

Total RNA was extracted from logarithmically growing ID8 cells (1×10^6 per dish) after 24 hours of incubation. The cells were then treated with different concentrations (0, 120, and 150 µM) of GA for 48 hours. Three samples were set for each concentration, and total RNA was extracted using Trizol reagent (15596026, Invitrogen, USA) according to the manufacturer’s instructions. Sequencing was performed by Beijing Novogene Bioinformatics Technology Co., Ltd.

### Differential gene expression analysis and GO, KEGG pathway enrichment analysis

The data were first analyzed for trends, followed by differential gene expression analysis using the DESeq2 algorithm. DEGs were identified based on the criteria of |log2(Fold Change)|≥1 and Padj ≤ 0.05. The DEGs were then subjected to Gene Ontology (GO) analysis and Kyoto Encyclopedia of Genes and Genomes (KEGG) functional enrichment analysis.

### Hoechst 33258 staining assay for apoptosis detection

Logarithmically growing A2780 and ES - 2 cells (1×10^5 per well) were seeded onto cover slips (placed in 6-well plates) and incubated for 24 hours. The medium was then replaced with complete medium containing different concentrations (0, 20, 40, and 60 µM) of GA and continued to be cultured for 48 hours. The cover slips were removed, fixed in 0.5 mL of fixative for 10 minutes, stained with 0.5 mL of Hoechst 33258 staining solution for 5 minutes, and mounted with antifade mounting medium on a slide. Apoptosis was observed under a fluorescence microscope (Nikon, Japan) and photographed.

### Annexin V-FITC/PI double staining for detection of apoptosis in A2780, ES - 2, and ID8 cells

The density of A2780, ES - 2, and ID8 cells was adjusted to 1.5×10^5 cells/mL and seeded in 6-well plates. After 24 hours, the medium was removed, and A2780 and ES - 2 cells were replaced with complete medium containing different concentrations (0, 20, 40, and 60 µM) of GA. ID8 cells were replaced with complete medium containing different concentrations (0, GA75, GA150, DDP5, GA75+DDP5, GA150+DDP5 µM) of GA. After 48 hours of incubation, the cells were washed with 4°C pre-cooled PBS (P010, Beijing Solarbio Science & Technology Co., Ltd., China), collected by centrifugation, and stained with Annexin V-FITC and PI according to the manufacturer’s instructions (556547, BD Biosciences, USA). The cells were analyzed using a CytoFlex flow cytometer (Beckman Coulter, USA) to detect apoptosis rates.

### Western blot analysis of apoptosis-, CXCL12/CXCR4 signaling pathway-, and PI3K/Akt/mTOR signaling pathway-related proteins in ovarian cancer cells

A2780 and ES - 2 cells treated with different concentrations of GA (0, 20, 40, 60 µM) and ID8 cells treated with different concentrations of GA (0, GA75, GA150, DDP5, GA75+DDP5, GA150+DDP5 µM) were lysed on ice with protein lysis buffer for 20 minutes. The lysates were centrifuged at 4°C, 12,000 rpm for 15 minutes, and the supernatants were collected to extract total protein. Protein concentration was determined using the BCA protein assay method. The protein samples were mixed with 5× protein loading buffer (P0015, Beyotime Institute of Biotechnology, China) and denatured at 99°C for 8 minutes. After electrophoresis and transfer to a membrane, the membrane was blocked and incubated with corresponding primary and secondary antibodies. The bands were visualized, and the relative expression levels were analyzed using ImageJ software.

### C57BL/6J mouse ovarian cancer xenograft model

#### Animal grouping and modeling

Forty-five healthy female C57BL/6J mice were acclimated for one week with standard diet and free access to water. Logarithmically growing Luc-ID8 cells transfected with the lucifemlrase gene were suspended in PBS to a concentration of 5×10^7 cells/. The mice were intraperitoneally injected with 0.1 mL of the cell suspension, and nine mice were randomly selected to begin treatment on the second day with intraperitoneal injection of gallic acid at a dose of 50 mg/kg every other day (12, 53). The remaining mice were subjected to *in vivo* imaging using an IVIS Lumina LT system (IVIS Lumina LT, USA) two weeks after modeling to observe abdominal tumor formation. The mice were then randomly divided into control (n=9), GA (50 mg/kg, n=9), DDP (2 mg/kg, n=9), and combination therapy (GA 50 mg/kg + DDP 2 mg/kg, n=9) groups. From the first treatment after tumor formation, the mice were treated every other day, and their body weight was measured. Imaging was performed every other day to monitor tumor size. On day 15 of treatment, three mice from each group were euthanized by cervical dislocation under isoflurane anesthesia, and tissues were collected. The remaining mice continued treatment until the endpoint was reached.

### HE staining for assessing the safety of GA on vital organs *in vivo*

The vital organs (heart, liver, spleen, lungs, kidneys, and brain) of the mice were fixed in 10% formalin, routinely dehydrated, embedded in paraffin, sectioned, and stained with HE. The pathological changes in the transplanted tumor tissues were observed under an optical microscope.

### Immunohistochemical staining for detection of Ki67 and cleaved-PARP protein expression levels

Paraffin sections were dewaxed with xylene, hydrated in ethanol, and repaired with pH 9.0 EDTA repair solution using high-pressure antigen retrieval. Non-specific staining was blocked with 3% hydrogen peroxide, and the sections were blocked with blocking solution. The sections were incubated with primary antibodies at room temperature for 1.5 hours, followed by secondary antibody incubation for 40 minutes, and stained with DAB. The sections were counterstained with hematoxylin, dehydrated, cleared, and mounted with glycerin gelatin mounting medium. The sections were dried at room temperature and observed under a microscope. The images were analyzed using ImageJ software. Tumor cells with brown-yellow nuclei were considered positive.

### Statistical analysis

Data analysis was performed using SPSS 20.0 statistical software. Quantitative data are presented as mean ± standard deviation (x ± s). Normality tests were conducted for all data, and homogeneity of variance tests were performed before comparing means among multiple groups. One-way analysis of variance was used for group comparisons, and LSD-t tests were used for pairwise comparisons between groups. *P*<0.05 was considered statistically significant.

## Data Availability

The datasets presented in this study can be found in online repositories. The names of the repository/repositories and accession number(s) can be found in the article/**Supplementary Material**.

## References

[1] Ghose A McCann L Makker S Mukherjee U Gullapalli S Erekkath J . Diagnostic biomarkers in ovarian cancer: advances beyond CA125 and HE4. Ther Adv Med Oncol. (2024) 16:1–12. doi: 10.1177/17588359241233225, PMID: 38435431 PMC10908239

[2] Ali AT Al-Ani O Al-Ani F . Epidemiology and risk factors for ovarian cancer. Menopausal Rev. (2023) 22:93–104. doi: 10.5114/pm.2023.128661, PMID: 37674925 PMC10477765

[3] Webb PM Jordan SJ . Global epidemiology of epithelial ovarian cancer. Nat Rev Clin Oncol. (2024) 21:389–400. doi: 10.1038/s41571-024-00881-3, PMID: 38548868

[4] Ronsini C Pasanisi F Greco P Cobellis L De Franciscis P Cianci S . Mininvasive cytoreduction surgery plus HIPEC for epithelial ovarian cancer: A systematic review. Med (Kaunas). (2023) 59:421. doi: 10.3390/medicina59030421, PMID: 36984422 PMC10055964

[5] Kobayashi Y Shimada M Tamate M Cho HW Zhu J Chou HH . Current treatment strategies for ovarian cancer in the East Asian Gynecologic Oncology Trial Group (EAGOT). J Gynecol Oncol. (2024) 35:e87. doi: 10.3802/jgo.2024.35.e87, PMID: 38606827 PMC11107282

[6] Atallah GA Kampan NC Chew KT Mokhtar NM Zin RRM Shafiee MNB . Predicting prognosis and platinum resistance in ovarian cancer: role of immunohistochemistry biomarkers. Int J Mol Sci. (2023) 24:1973. doi: 10.3390/ijms24031973, PMID: 36768291 PMC9916805

[7] Verleye L Castanares-Zapatero D Devos C De Gendt C Silversmit G Van Damme N . Survival in stage IV ovarian cancer with increased use of debulking surgery and bevacizumab. Int J Gynecol Cancer. (2023) 33:543–8. doi: 10.1136/ijgc-2022-003813, PMID: 36604121

[8] Cao W Chen HD Yu YW Li N Chen WQ . Changing profiles of cancer burden worldwide and in China: a secondary analysis of the global cancer statistics 2020. Chin Med J. (2021) 134:783–91. doi: 10.1097/CM9.0000000000001474, PMID: 33734139 PMC8104205

[9] Borsoi FT Arruda HS Andrade AC Dos Santos MP da Silva IN Marson LA . Araçá-boi extract and gallic acid reduce cell viability and modify the expression of tumor suppressor genes and genes involved in epigenetic processes in ovarian cancer. Plants (Basel):1671. (2025) 14. doi: 10.3390/plants14111671, PMID: 40508345 PMC12158145

[10] He Z Chen AY Rojanasakul Y Rankin GO Chen YC . Gallic acid, a phenolic compound, exerts anti-angiogenic effects via the PTEN/AKT/HIF-1α/VEGF signaling pathway in ovarian cancer cells. Oncol Rep. (2016) 35:291–7. doi: 10.3892/or.2015.4354, PMID: 26530725 PMC4699619

[11] Sánchez-Carranza JN Díaz JF Redondo-Horcajo M Barasoain I Alvarez L Lastres P . Gallic acid sensitizes paclitaxel-resistant human ovarian carcinoma cells through an increase in reactive oxygen species and subsequent downregulation of ERK activation. Oncol Rep. (2018) 39:3007–14. doi: 10.3892/or.2018.6382, PMID: 29693189

[12] Varela-Rodríguez L Sánchez-Ramírez B Hernández-Ramírez VI Varela-Rodríguez H Castellanos-Mijangos RD González-Horta C . Effect of Gallic acid and Myricetin on ovarian cancer models: a possible alternative antitumoral treatment. BMC Complement Med Ther. (2020) 20:110. doi: 10.1186/s12906-020-02900-z, PMID: 32276584 PMC7149887

[13] Dasari S Njiki S Mbemi A Yedjou CG Tchounwou PB . Pharmacological effects of cisplatin combination with natural products in cancer chemotherapy. Int J Mol Sci. (2022) 23:1532. doi: 10.3390/ijms23031532, PMID: 35163459 PMC8835907

[14] Strik H Efferth T Kaina B . Artesunate in glioblastoma therapy: Case reports and review of clinical studies. Phytomedicine. (2024) 123:155274. doi: 10.1016/j.phymed.2023.155274, PMID: 38142662

[15] Fang SH Plesa M Carchman EH Cowell NA Staudt E Twaroski KA . A phase I study of intra-anal artesunate (suppositories) to treat anal high-grade squamous intraepithelial lesions. PloS One. (2023) 18:e0295647. doi: 10.1371/journal.pone.0295647, PMID: 38100463 PMC10723659

[16] Khaiwa N Maarouf NR Darwish MH Alhamad DWM Sebastian A Hamad M . Camptothecin’s journey from discovery to WHO Essential Medicine: Fifty years of promise. Eur J Med Chem. (2021) 223:113639. doi: 10.1016/j.ejmech.2021.113639, PMID: 34175539

[17] Khan AQ Ahmed EI Elareer N Fathima H Prabhu KS Siveen KS . Curcumin-mediated apoptotic cell death in papillary thyroid cancer and cancer stem-like cells through targeting of the JAK/STAT3 signaling pathway. Int J Mol Sci. (2020) 21:438. doi: 10.3390/ijms21020438, PMID: 31936675 PMC7014270

[18] Hsu WH Hua WJ Qiu WL Tseng AJ Cheng HC Lin TY . WSG, a glucose-enriched polysaccharide from Ganoderma lucidum, suppresses tongue cancer cells via inhibition of EGFR-mediated signaling and potentiates cisplatin-induced apoptosis. Int J Biol Macromol. (2021) 193:1201–8. doi: 10.1016/j.ijbiomac.2021.10.146, PMID: 34742847

[19] Howes M-JR . The evolution of anticancer drug discovery from plants. Lancet Oncol. (2018) 19:293–4. doi: 10.1016/S1470-2045(18)30136-0, PMID: 29508748

[20] Keyvani-Ghamsari S Rahimi M Khorsandi K . An update on the potential mechanism of gallic acid as an antibacterial and anticancer agent. Food Sci Nutr. (2023) 11:5856–72. doi: 10.1002/fsn3.3615, PMID: 37823155 PMC10563697

[21] Jiang Y Pei J Zheng Y Miao YJ Duan BZ Huang LF . Gallic acid: A potential anti-cancer agent. Chin J Integr Med. (2022) 28:661–71. doi: 10.1007/s11655-021-3345-2, PMID: 34755289

[22] Ashrafizadeh M Zarrabi A Mirzaei S Hashemi F Samarghandian S Zabolian A . Gallic acid for cancer therapy: Molecular mechanisms and boosting efficacy by nanoscopical delivery. Food Chem Toxicol. (2021) 157:112576. doi: 10.1016/j.fct.2021.112576, PMID: 34571052

[23] Moghtaderi H Sepehri H Delphi L Attari F . Gallic acid and curcumin induce cytotoxicity and apoptosis in human breast cancer cell MDA-MB-231. BioImpacts: BI. (2018) 8:185–94. doi: 10.15171/bi.2018.21, PMID: 30211078 PMC6128975

[24] Zhang T Ma L Wu P Li W Li T Gu R . Gallic acid has anticancer activity and enhances the anticancer effects of cisplatin in non−small cell lung cancer A549 cells via the JAK/STAT3 signaling pathway. Oncol Rep. (2019) 41:1779–88. doi: 10.3892/or.2019.6976, PMID: 30747218

[25] Yazgan Y Cinar R . Gallic acid enhances cisplatin-induced death of human laryngeal cancer cells by activating the TRPM2 channel. Dokl Biochem Biophys. (2025) 521:221–31. doi: 10.1134/S1607672924601276, PMID: 40216719

[26] Zeng M Su Y Li K Jin D Li Q Li Y . Gallic acid inhibits bladder cancer T24 cell progression through mitochondrial dysfunction and PI3K/akt/NF-κB signaling suppression. Front Pharmacol. (2020) 11:1222. doi: 10.3389/fphar.2020.01222, PMID: 32973496 PMC7468429

[27] He Z Liu X Wu F Wu S Rankin GON Martinez I . Gallic acid induces S and G2 phase arrest and apoptosis in human ovarian cancer cells *in vitro*. Appl Sci (Basel). (2021) 11:3807. doi: 10.3390/app11093807, PMID: 34386269 PMC8356902

[28] Nakatomi T Itaya-Takahashi M Horikoshi Y Shimizu N Parida IS Jutanom M . The difference in the cellular uptake of tocopherol and tocotrienol is influenced by their affinities to albumin. Sci Rep. (2023) 13:7392. doi: 10.1038/s41598-023-34584-z, PMID: 37149706 PMC10164177

[29] Varghese R Dalvi YB . Natural products as anticancer agents. Curr Drug Targets. (2021) 22:1272–87. doi: 10.2174/1389450121999201230204526, PMID: 33390130

[30] Dong M Shan B Han X Zhao X Wang F Zhu L . Baseline mutations and up-regulation of PI3K-AKT pathway serve as potential indicators of lack of response to neoadjuvant chemotherapy in stage II/III breast cancer. Front Oncol. (2021) 11:784985. doi: 10.3389/fonc.2021.784985, PMID: 35480699 PMC9036956

[31] Jin Y Chen Y Tang H Hu X Hubert SM Li Q . Activation of PI3K/AKT pathway is a potential mechanism of treatment resistance in small cell lung cancer. Clin Cancer Res. (2022) 28:526–39. doi: 10.1158/1078-0432.CCR-21-1943, PMID: 34921019

[32] Di J Bo W Wang C Liu C . Ailanthone increases cisplatin-induced apoptosis and autophagy in cisplatin resistance non-small cell lung cancer cells through the PI3K/AKT/mTOR pathway. Curr Med Chem. (2024) 32(33):7357–76. doi: 10.2174/0109298673315460240816091032, PMID: 39192653

[33] Sandhiutami NMD Arozal W Louisa M Rahmat D Wuyung PE . Curcumin nanoparticle enhances the anticancer effect of cisplatin by inhibiting PI3K/AKT and JAK/STAT3 pathway in rat ovarian carcinoma induced by DMBA. Front Pharmacol. (2021) 11. doi: 10.3389/fphar.2020.603235, PMID: 33536913 PMC7848208

[34] Jiang T Liang Y Ji Y Xue Y . Fisetin enhances cisplatin sensitivity in renal cell carcinoma via the CDK6/PI3K/Akt/mTOR signaling pathway. Oncol Lett. (2024) 27:165. doi: 10.3892/ol.2024.14298, PMID: 38426151 PMC10902757

[35] Yang CH Chang YC Hsu CC Lin CH Chen IJ Wu YT . Fucoidan enhances cisplatin-induced effects on SCC - 25 human oral cancer cells by inhibiting the PI3K/AKT pathway. Anticancer Res. (2023) 43:4015–22. doi: 10.21873/anticanres.16589, PMID: 37648299

[36] Lin J Song T Li C Mao W . GSK - 3β in DNA repair, apoptosis, and resistance of chemotherapy, radiotherapy of cancer. Biochim Biophys Acta Mol Cell Res. (2020) 1867:118659. doi: 10.1016/j.bbamcr.2020.118659, PMID: 31978503

[37] Beurel E Grieco SF Jope RS . Glycogen synthase kinase-3 (GSK3): regulation, actions, and diseases. Pharmacol Ther. (2015) 148:114–31. doi: 10.1016/j.pharmthera.2014.11.016, PMID: 25435019 PMC4340754

[38] Meng W Xue S Chen Y . The role of CXCL12 in tumor microenvironment. Gene. (2018) 641:105–10. doi: 10.1016/j.gene.2017.10.015, PMID: 29017963

[39] Teicher BA Fricker SP . CXCL12 (SDF - 1)/CXCR4 pathway in cancer. Clin Cancer Res. (2010) 16:2927–31. doi: 10.1158/1078-0432.CCR-09-2329, PMID: 20484021

[40] Mortezaee K . CXCL12/CXCR4 axis in the microenvironment of solid tumors: A critical mediator of metastasis. Life Sci. (2020) 249:117534. doi: 10.1016/j.lfs.2020.117534, PMID: 32156548

[41] Yin X Xia K Peng S Tan B Huang Y Wang M . ABCF1/CXCL12/CXCR4 enhances glioblastoma cell proliferation, migration, and invasion by activating the PI3K/AKT signal pathway. Dev Neurosci. (2023) 46(3):210–20. doi: 10.1159/000533130, PMID: 37757768

[42] Glaviano A Foo ASC Lam HY Yap KCH Jacot W Jones RH . PI3K/AKT/mTOR signaling transduction pathway and targeted therapies in cancer. Mol Cancer. (2023) 22:138. doi: 10.1186/s12943-023-01827-6, PMID: 37596643 PMC10436543

[43] Adams KM Wendt JR Wood J Olson S Moreno R Jin Z . Cell-intrinsic platinum response and associated genetic and gene expression signatures in ovarian cancer. Cancer Gene Ther. (2025) 32(9):985–96. doi: 10.1101/2024.07.26.605381, PMID: 40683954 PMC12396967

[44] Zhou J Le K Xu M Ming J Yang W Zhang Q . CXCR4 antagonist AMD3100 reverses the resistance to tamoxifen in breast cancer via inhibiting AKT phosphorylation. Mol Ther Oncol. (2020) 18:161–70. doi: 10.1016/j.omto.2020.06.009, PMID: 32691010 PMC7311345

[45] Liu S Xie SM Liu W Gagea M Hanker AB Nguyen N . Targeting CXCR4 abrogates resistance to trastuzumab by blocking cell cycle progression and synergizes with docetaxel in breast cancer treatment. Breast Cancer Res. (2023) 25:62. doi: 10.1186/s13058-023-01665-w, PMID: 37280713 PMC10245436

[46] Han X Gao Y Jiang M Li Z Guo J Li Y . Single-cell and spatial transcriptome sequencing uncover a platinum-resistant cluster overexpressed TACSTD2 in high-grade serous ovarian cancer. J Cancer. (2024) 15:3427–40. doi: 10.7150/jca.95269, PMID: 38817863 PMC11134433

[47] Guocai X Dongyan W Huaiwu L Zhongqiu L Bingzhong Z . Resistance reversion of rapamycin on ovarian cancer cell lineSKOV3/DDP andItslolecularMechanisms. J Sun Yat-sen Univ (Medical Edition). (2018) 39:68–72.

[48] Choubey S Goyal S Varughese LR Kumar V Sharma AK Beniwal V . Probing gallic acid for its broad spectrum applications. Mini Rev Med Chem. (2018) 18:1283–93. doi: 10.2174/1389557518666180330114010, PMID: 29600764

[49] Harishkumar R Reddy LPK Karadkar SH Murad MA Karthik SS Manigandan S . Toxicity and selective biochemical assessment of quercetin, gallic acid, and curcumin in zebrafish. Biol Pharm Bull. (2019) 42:1969–76. doi: 10.1248/bpb.b19-00296, PMID: 31787712

[50] Chou T-C . Theoretical basis, experimental design, and computerized simulation of synergism and antagonism in drug combination studies. Pharmacol Rev. (2006) 58:621–81. doi: 10.1124/pr.58.3.10, PMID: 16968952

[51] You BR Park WH . Gallic acid-induced lung cancer cell death is related to glutathione depletion as well as reactive oxygen species increase. Toxicol In Vitro. (2010) 24:1356–62. doi: 10.1016/j.tiv.2010.04.009, PMID: 20417267

[52] Doncheva NT Morris JH Gorodkin J Jensen LJ . Cytoscape stringApp: network analysis and visualization of proteomics data. J Proteome Res. (2019) 18:623–32. doi: 10.1021/acs.jproteome.8b00702, PMID: 30450911 PMC6800166

[53] Jang Y-G Ko E-B Choi K-C . Gallic acid, a phenolic acid, hinders the progression of prostate cancer by inhibition of histone deacetylase 1 and 2 expression. J Nutr Biochem. (2020) 84:108444. doi: 10.1016/j.jnutbio.2020.108444, PMID: 32615369

