# Supplementary material for: Gallic acid potentiates the anticancer efficacy of cisplatin in ovarian cancer cells through modulation of the PI3K/AKT/mTOR and CXCL12/CXCR4 signaling pathways
Source: Front Oncol. 2025 Sep 22;15:1653538. doi: 10.3389/fonc.2025.1653538 (PMC12497589; doi:10.3389/fonc.2025.1653538)
Supplement: Supplementary file 1 [file DataSheet1.docx]

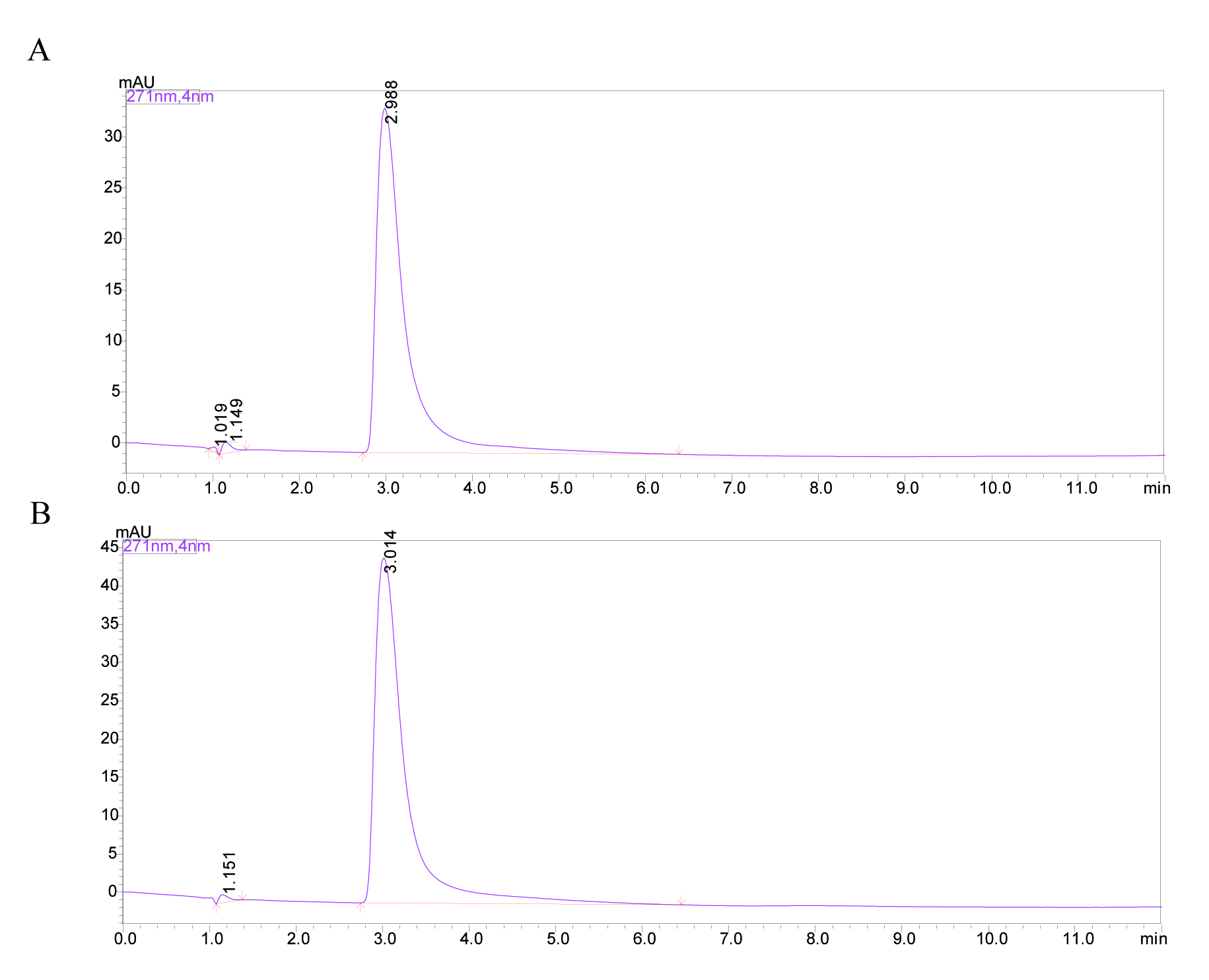


S1. Gallicacidpuritydetermination. A. Gallic acid standard HPLC; B. YMSZS HPLC

Table S1 Purity determination results

| Sample  (mg) | Retention time | Peak area | （mg/mg） | Purity(%) | Average valuue(%) |
| --- | --- | --- | --- | --- | --- |
| 0.26 | 3.004 | 252693 | 0.9713 | 97.13 | 98.34 |
|  | 3.001 | 257993 | 0.9902 | 99.02 |  |
|  | 2.994 | 257618 | 0.9888 | 98.88 |  |
